# From Global to Nano: A Geographical Perspective of *Aggregatibacter actinomycetemcomitans*

**DOI:** 10.3390/pathogens13100837

**Published:** 2024-09-27

**Authors:** Mark I. Ryder, Daniel H. Fine, Annelise E. Barron

**Affiliations:** 1 Department of Bioengineering, School of Medicine and School of Engineering, Stanford University, Stanford, CA 94143, USA; aebarron@stanford.edu; 2 Division of Periodontology, Department of Orofacial Sciences, School of Dentistry, University of California San Francisco, San Francisco, CA 94143, USA; 3 Department of Oral Biology, Rutgers School of Dental Medicine, 443 Via Ortega, Stanford, CA 94305, USA

**Keywords:** *Aggregatibacter actinomycetemcomitans*, aggressive periodontitis, *Porphyromonas gingivalis*, biogeography, biofilms, Localized Stage III Grade C periodontitis

## Abstract

The periodontal disease pathobiont *Aggregatibacter actinomycetemcomitans* (*A. actinomycetemcomitans*) may exert a range of detrimental effects on periodontal diseases in general and, more specifically, with the initiation and progression of Localized Stage III Grade C periodontitis (molar–incisor pattern). In this review of the biogeography of this pathobiont, the full range of geographical scales for *A. actinomycetemcomitans*, from global origins and transmission to local geographical regions, to more locally exposed probands and families, to the individual host, down to the oral cavity, and finally, to spatial interactions with other commensals and pathobionts within the plaque biofilms at the micron/nanoscale, are reviewed. Using the newest technologies in genetics, imaging, in vitro cultures, and other research disciplines, investigators may be able to gain new insights to the role of this pathobiont in the unique initial destructive patterns of Localized Stage III Grade C periodontitis. These findings may incorporate the unique features of the microbiome that are influenced by variations in the geographic environment within the entire mouth. Additional insights into the geographic distribution of molar–incisor periodontal breakdown for Localized Stage III Grade C periodontitis may derive from the spatial interactions between *A. actinomycetemcomitans* and other pathobionts such as *Porphyromonas gingivalis*, *Filifactor aclocis*, and commensals such as *Streptococcus gordonii*. In addition, while the association of *A. actinomycetemcomitans* in systemic diseases is limited at the present time, future studies into possible periodontal disease–systemic disease links may also find *A. actinomycetemcomitans* and its geographical interactions with other microbiome members to provide important clues as to implications of pathobiological communications.

## 1. Introduction

The series of papers in this monograph present an extensive review of the range of pathological effects of *Aggregatibacter actinomycetemcomitans* (*A. actinomycetemcomitans*), a periodontal pathobiont, with the focus on Localized Stage III Grade C periodontitis (AKA Localized Molar–Incisor Grade III Stage C periodontitis, Localized Juvenile periodontitis, or Localized Aggressive periodontitis). The role of this pathobiont through its prevalence in dysbiotic microbial communities, suppression effects on the host response, toxicity to periodontal tissues, and stimulation of the destructive arms of the inflammatory response has been extensively investigated [1]. The arsenal of destructive weapons of *A. actinomycetemcomitans* include toxins within the fimbriae on the surface of the bacteria, secreted cytolethal distending toxins, and lipopolysaccharides [1]. These detrimental effects of *A. actinomycetemcomitans* can be modified in periodontal diseases on the smallest geographical scale by the spatial proximity to other periodontal pathobionts, as well as commensal bacteria [2]. In addition, when discussing the broader definition of geography for a pathobiont such as *A. actinomycetemcomitans*, one can expand the range of scales from the largest origins and transmission on a global scale to local geographical regions, to more locally exposed probands, and to within families. From a geographical perspective, we can then consider the oral cavity as a whole, the plaque biofilm structure, and finally. at the smallest scale of individual interactions between *A. actinomycetemcomitans* and adjacent or nearby commensal organisms and pathobionts at the sub-micron/nanoscale in supragingival, subgingival, and systemically affected environments.

In this geographical tour of *A. actinomycetemcomitans* and Localized Stage III Grade C periodontitis, we start at these largest global scales and work down to the smallest nanometer scales to address how this pathobiont may play a key role to this rapidly progressing form of periodontal disease, as well as in the exacerbation of systemic diseases. Studies of the geography of *A. actinomycetemcomitans* may help answer central questions regarding the ethnic, racial, and familial distributions of this rapidly progressing periodontal disease, as well as the specific intraoral geographical location of this disease to first molars and incisors. While some of these questions on the global to nanoscale remain unanswered, and theories arising from published observations are yet to be confirmed, a broad range of techniques used in these approaches described in this review may yield new insights into these global to nanoscale geographical questions.

## 2. Global Origins

In examining geography at the “widest field of vision”, a variety of genetic tools have been used to trace the global migration of the potential geographic origins of this pathobiont, as well as the divergence of this pathobiont from the species level to serotypes and strains [3,4]. Initially, genetic signatures have been employed to determine both the common origin and routes of dissemination and divergence [5]. Aside from microbiology, there are interesting parallels of such approaches in fields of study such as epidemiology, anthropology, genetics, and even in disciplines as far afield as linguistics [6].

Geographical concepts can be addressed at the broad cultural level that can include language and anatomical appearances. The broader cultural and biological structures of language and gross anatomical appearance can then be assessed at a finer level of resolution using genomic data [7]. For example, in order to determine the geographic origin for the migration patterns of the Polynesian people, both linguists and human and animal anatomists and geneticists have used the “language tools” of their respective disciplines to propose that the origin of these migrations were from early civilizations from the southern tip of Taiwan [8,9]. Similarly, using the genetics of primate mitochondria, anthropologists working with geneticists have postulated a “Mitochondrial Eve” originating in defined areas of East Africa as the geographic origin of migrating *Homo sapiens* populations [10]. By analogy to this type of geographical genetic tracing, we can draw interesting parallels to the more rapid evolution, divergence, and migration patterns of SARS-CoV-2 strains [11]. In particular, the emergence of a particular SARS-CoV-2 strain variant and its initial predominance and probable origins from South Africa, then spreading to more northern locations in the African continent, has been demonstrated using these tools [12].

**Figure 1:**
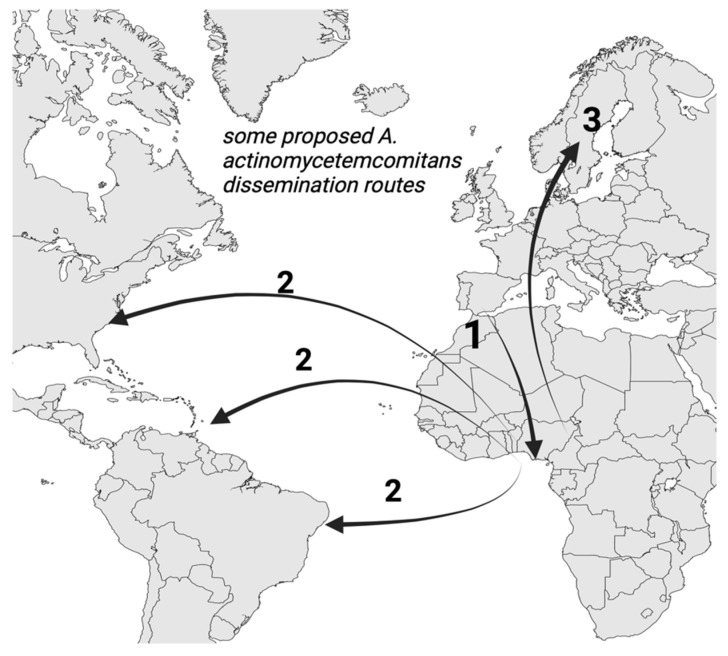
A proposed geographical origin and pathway of dissemination of *A. actinomycetemcomitans* in the African American population through genetic back tracing: 1. Origin from North Africa to West Central Africa and then to 2. North and South America through the slave trade [14,15,16]. 3. Regarding the spread to other parts of the world where *A. actinomycetemcomitans* may have disseminated and been detected, these geographical routes have yet to be determined but may include discrete geographic zones, such as regions of Sweden [16,17] (original image created by the author for this manuscript with Biorender.com).

For the geographic origins of *A. actinomycetemcomitans*, similar back tracing approaches have determined in part why the spread of this pathobiont is more common in African American populations [13]. For example, studies restricted to limited regions such as Africa and Scandinavia and analysis of clones prevalent in these populations indicate that the origin of one or more of the more pathogenic strains of *A. actinomycetemcomitans* (the JP 2 clone) may have been in Mediterranean Africa [14] around present day Morocco; after which, it spread to Western Africa and then to the Americas, due in part to migration patterns of the slave trade [15] (Figure 1). In Scandinavia, the migration patterns may have resulted in the presence of the JP 2 clone in smaller, discrete geographical areas. For example, in two studies in Sweden, this pathogenic clone was detected in two studies [16,17], while, in Denmark, a study of younger subjects did not detect the prevalence of this clone [18].

## 3. Smaller Populations and the Individual

At a higher magnification of the geographical level, it is well established that cases of Localized Stage III Grade C periodontitis cluster within certain racial/ethnic groups and within families [13,19]. In addition to the local exposure from close geographical proximity, a genetic component may play an important role in this pattern [19]. From a microbiological perspective, the roles of both genetics and host response elements raise questions as to whether *A. actinomycetemcomitans* transmission occurs primarily within the smaller physical spaces of families or whether geographically adjacent probands may also play a role.

The use of various genetic fingerprinting techniques have demonstrated that there is strong evidence for the physical/geographical transmission of *A. actinomycetemcomitans* within the closer physical confines of families, even to members that do not show evidence of the disease [20]. While a developing fetus may not be expected to acquire *A. actinomycetemcomitans* in the womb, the initial passage of the child through the birth canal can potentially expose the child first to the mother’s microbiome [21,22]. While prior studies have not demonstrated *A. actinomycetemcomitans* per se in the birth canal pathway, other periodontal pathobionts have been detected in these anatomical regions [23]. Nevertheless, acquisition of *A. actinomycetemcomitans* as a risk factor in the initiation of localized Stage III Grade C periodontitis [24,25,26,27] could occur from mother to child immediately postpartum with exposure to the close geographic environment [28]. Evidence for this earlier physical acquisition of *A. actinomycetemcomitans* before clinical signs of this rapid form of periodontitis is supported by studies that have shown similar patterns of rapid destruction in the primary teeth in the same geographical areas of the mouth as the future affected first molars [29]. Once established within a closely geographically associated proband such as family or community, there may be the retention of specific species and strains of pathogenic microbiota such as *A. actinomycetemcomitans* and *Porphyromonas gingivalis* (*P. gingivalis*) [20]. Evidence to support this persistence of specific microbial profiles of periodontal pathobionts in the form of dysbiotic communities has been confirmed from early studies that demonstrated the retention of the same species and strains of species of periodontal pathobionts [30], including *P. gingivalis*, as well as *A. actinomycetemcomitans*, even after attempts at debridement and maintenance [31,32], and since these pathobionts may reside in multiple inaccessible geographical areas in the mouth, their complete elimination from the oral cavity is not possible. Therefore, there is a risk of recolonization into their originally colonized intraoral geographical areas.

One of the most extensively studied areas in dental and medical research is the potential for periodontal pathobionts to translocate to other parts of the body and thereby induce both local inflammatory and destructive responses, as well as an elevated state of systemic inflammation. These pathways of microbial translocation include invasion of pathobionts into the bloodstream and lymphatic system through the breakdown of the epithelial barrier between the gingival tissue and sulcus microbiota spread along neural pathways, and direct invasion of pathobionts into tissue [33,34,35]. In addition, periodontal pathobionts can directly invade host cells such as epithelial cells and immune cells in the periodontal tissue and then be carried as “Trojan horses” to distal sites of the body [36]. This proposed translocation pathway mechanism has been extensively studied and observed for the periodontal pathobiont *P. gingivalis* [34,36]. For both *A. actinomycetemcomitans* and *P. gingivalis*, epithelial cell mechanisms for adhesion and rearranging the actin cytoskeleton of the host cell function have been shown to facilitate ingestion into the cell [37].

To date, there has been some published evidence that has focused on the clinical implications of the geographic translocation of *A. actinomycetemcomitans* to other parts of the body. Individual case reports or series of case reports have noted the presence of *A. actinomycetemcomitans* in various pulmonary infections [38], non-oral abscesses [39], and general septicemia. For *A. actinomycetemcomitans*, the most studied of these non-oral infections to distal sites in the body is for cardiovascular disease—in particular, endocarditis [40,41,42] and atherosclerosis [43]. The detection rates of this pathobiont in endocarditis have been reported to range from 0.6% for *A. actinomycetemcomitans* per se to 3% for pathobionts in a related group of eight species found in periodontal disease plaques (HACEK group) [5] that may include *A. actinomycetemcomitans.* These apparently low rates for *A. actinomycetemcomitans* detection from these earlier studies may actually be higher when using newer big data and high-throughput approaches with more detailed and sensitive 16S ribotyping techniques [44,45,46]. This is particularly relevant because *A. actinomycetemcomitans* is not as elevated in subgingival plaque and, in general, is more prevalent in specific segments of the population [47,48]. In addition, since the new World Workshop Classification meeting in 2017, there has been a highly significant decrease in the ratio of publications that feature *A. actinomycetemcomitans* as compared to *P. gingivalis*. Importantly, an extensive series of case reports provided evidence that *A. actinomycetemcomitans* can translocate to a variety of organs [39]. More specifically, an extensive review of 26 studies related to the recovery of oral pathobionts from atherosclerotic cardiovascular samples has shown 8 out of 26 where *P. gingivalis* recovery was the most prevalent, whereas 7 studies showed *A. actinomycetemcomitans* to be the most prevalent [43]. None of the other six reputed pathobionts reached a higher level than *A. actinomycetemcomitans* or *P. gingivalis* in any samples taken [43]. Furthermore, in addition to the potential translocation of whole *A. actinomycetemcomitans*, mouse models have shown that *A. actinomycetemcomitans*, like the more extensively studied *P. gingivalis* [49], can secrete outer membrane vesicles (OMV’s) that can potentially cross the blood–brain barrier and induce neuroinflammation [36,50,51]. Whether this phenomenon of OMV’s from *A. actinomycetemcomitans* also exists in humans has yet to be investigated. As demonstrated in previous studies with OMVs from *P. gingivalis* [34,52], if such a translocation for *A. actinomycetemcomitans* does occur, it may have broad implications for the initiation and progression of Alzheimer’s disease and other forms of dementia. It should, however, be noted that, unlike the increased levels of the detection of *P. gingivalis* in the periodontal disease microbiome in older adults, it appears that levels of *A. actinomycetemcomitans* may decrease in the periodontal disease microbiome in older adults [53]. Nevertheless, it is possible that any translocation of a periodontal pathobiont at an earlier age may contribute to the upstream chain of events that may become clinically detectable diseases and conditions later in life.

## 4. The Oral Cavity

These aforementioned examples provide evidence for the geographic origins, transmission, and strain diversity of *A. actinomycetemcomitans* on the global, familial, and whole individual scales. When we refocus at the smaller geographical scale on the central role of *A. actinomycetemcomitans* in Localized Stage III grade C periodontitis, a central question arises as follows: Since this condition is initially localized to the region of the first molars and incisor teeth in the permanent dentition and corresponding areas of the primary dentition, can the local microbial geography and ecology of the mouth explain, in part, the initial localization and rapid bone loss in the first molar and incisor regions in both the primary and permanent dentition? These local geographic factors within the mouth can interact with the larger scale systemic variations and risk factors such as variations in the systemic host response to *A. actinomycetemcomitans*. These overarching factors are presented in other papers of this monograph.

**Figure 2:**
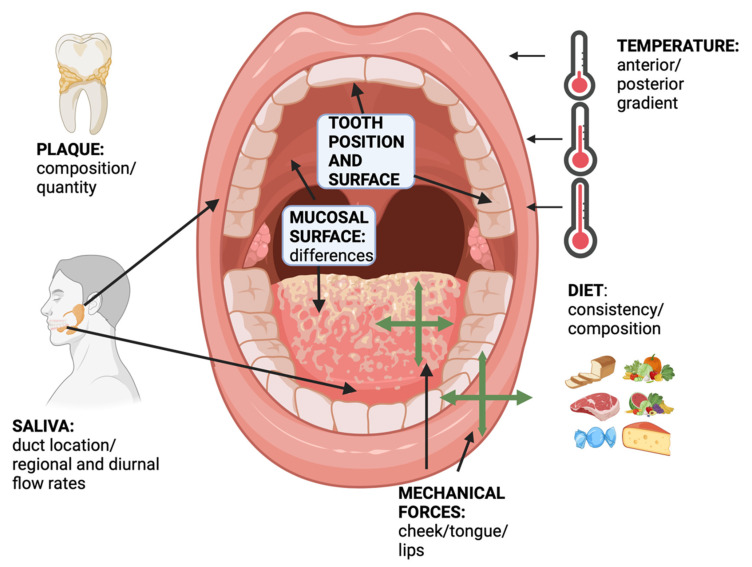
Differences in physical geography of the oral cavity that may affect regional variations of the location and distribution of *A. actinomycetemcomitans* may include tooth location, tooth type, and tooth surface; temperature gradients from the anterior to posterior oral cavity; attachment to different mucosal surfaces; location of the salivary ducts, salivary composition, and differences in salivary flow; effects of the cheek, tongue, and masticatory musculature; regional variations in the quantity of plaque accumulation; and chemical and physical characteristics of the diet (original image created by the author for this manuscript with Biorender.com).

Over the past several decades, there have been investigations into the influence of regional variations of the conditions in the mouth that may affect regional differences in the microbiome, which have been taken into consideration: (1) the location of the microbiome by tooth type and position in the mouth [54,55]; (2) temperature gradients within the oral cavity [56]; (3) soft tissue type and surface; (4) location of the salivary glands and overall levels of salivary flow, which may have both diurnal variation and variation before, during, and after mastication and in hyposalivation conditions such as Sjogren’s syndrome [57]; (5) differences in the levels of salivary flow from the front and back of the mouth and around salivary duct openings [58]; (6) physical effects of mastication, including forces of the musculature of the cheek and tongue on the teeth during periods of mastication and periods where the mastication of food is not occurring; (7) differences in the plaque mass per se around teeth in different regions of the mouth [59]; and (8) the chemical nature and physical consistency of their diet [44,57,60,61,62] (Figure 2).

In a review by Proctor et al. [60], the principles that may govern the regional distribution and profile of the periodontopathic microflora within a small geographic niche were more extensively reviewed using the principles of selection, physical dispersal, and genetic drift within an individual species and increased or decreased species diversity [60]. There have been several studies that demonstrated a preferential colonization and high recovery rate of *A. actinomycetemcomitans* in the first molar regions both with and without rapid periodontal breakdown at the time of sample collection [63,64,65]. One early study by Mombelli et al. demonstrated a high predictive rate of the presence of *A. actinomycetemcomitans* on a first mandibular molar on one side of the mouth and the presence of *A. actinomycetemcomitans* on the contralateral first mandibular molar, thereby supporting the concept of bilateral symmetry of microbial profiles in periodontal disease [66]. Furthermore, Haffajee et al. demonstrated that, when compared to the broader distribution *of P. gingivalis* in a wider area of the dentition of periodontal diseases, *A. actinomycetemcomitans* was detected in more limited locations, such as the first molar region [67]. These detection rates for *A. actinomycetemcomitans* are seen in more discrete areas as constituents of complex supragingival and subgingival biofilms in contrast to other surfaces of the oral cavity, which may be explained in part by a relatively smaller proportion of *A. actinomycetemcomitans* in these complex biofilms. These findings could be due to older, less sensitive culturing techniques or DNA checkboard techniques [68].

However, using new bioinformatics and big data approaches, future studies that characterize the microbiome on a tooth-by-tooth and site-by-site basis for both supra and subgingival biofilms in deciduous, mixed, and permanent dentitions, which show the clinical hallmarks of Localized Stage III grade C patients, may shed light on the influence of the eight factors listed above on the geographic variations in the total microbial environment. In addition, eruption patterns of the permanent teeth could be another factor for *A. actinomycetemcomitans* localization [69]. The new technologies could lead to an increased understanding of the presence and/or pathogenic potential of *A. actinomycetemcomitans* to these molar/incisor sites. Furthermore, comparing these distributions with the tooth-by-tooth microbial profiles of periodontally healthy subjects and with subjects with less severe periodontitis in Stage I or II/Grade A or B classes may further our understanding of the unique localized periodontal breakdown patterns in subjects with Localized Stage III Grade C periodontitis.

The promise of these approaches that focus on the site-by-site location of bacterial species and distribution patterns for the entire mouth is supported by recent work [57,60,61] designed to examine the effects of hyposalivation on gradients in the patterns, diversity, and spatial connections of the microbiome. These approaches have included the collection of supragingival samples from each buccal and lingual tooth surface, selected subgingival samples, and intraoral soft tissue surface sites including exfoliated epithelial cells (which may serve as an early reservoir for *A. actinomycetemcomitans* [70]). These investigations have shown different gradients in phyla distribution with different corresponding levels of salivary flow and strong similarities in phyla distribution based on closer geographical distancing as opposed to inter-arch or bilateral geographical distances. From these observations on phyla and diversity distributions based on salivary flow, such distribution mapping may aid researchers and clinicians in identifying patients at risk for caries and periodontal diseases. By using these tools at finer resolution to the genus, species, and strain levels, investigators may also gain new insights into the next higher geographical magnification of different tooth environments that can identify interactions of *A. actinomycetemcomitans* with the total local microflora in individual sulcus sites. These tools could be used to study the biofilm adherent to the tooth surface, the overlying planktonic suspension of bacteria in the gingival sulcus, local invasion of bacteria into the periodontal tissues and within tissue, and host response cells within the tissue. The promise of studying such interactions using the approaches described in this section can examine this microbial geography at the highest resolution.

## 5. Microbial Communities and Biofilms

There is compelling evidence that the survival and pathogenic potential of *A. actinomycetemcomitans* is dependent on the local interactions of this pathobiont with the complex communities of both the supra gingival and sub gingival biofilms. These interactions occur geographically on the micro and nanoscales and include interactions with microbial extracellular matrices, quorum sensing, exchange of genetic material, exchange of nutrients, and neutralization of host defense molecules [71,72,73]. While extensive studies have been published on such interactions to the whole biofilm for individual pathobiont species such as *P. gingivalis*, *T. denticola*, and *Fusobacterium nucleatum* (*F. nucleatum*), there is a dearth of studies that focused on *A. actinomycetemcomitans* from this high magnification geographical viewpoint. Such studies are important, as sampling studies from sites with Localized Stage III Grade C periodontitis have shown recovery rates of *A. actinomycetemcomitans* with other periodontal pathobionts such as *Filofactor alocis* (*F. alocis*) and *P. gingivalis* [74]. Two newer approaches that take into account the geographical proximity of *A. actinomycetemcomitans* to these other pathobionts have the potential to yield new insights into the pathogenic role of this species in these Localized Stage III grade C lesions [75,76].

**Figure 3:**
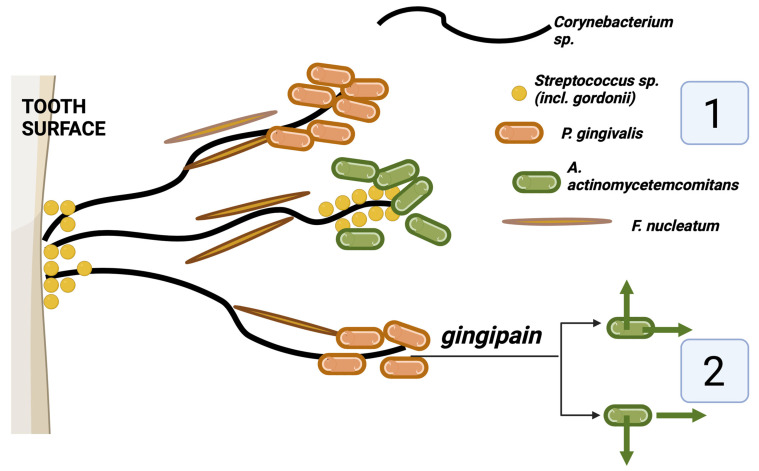
A proposed geographical model distribution of *A. actinomycetemcomitans* in the plaque biofilm based on diverse prior imaging studies and in vitro studies [76,81,82,83]. (**1**) *A. actinomycetemcomitans* may cluster around streptococcus “corncob” formations at the end of the filamentous corynebacterium species and may also form clusters near other pathobionts such as *P. gingivalis*. (**2**) However, gingipain enzymes from *P. gingivalis* may also exclude *A. actinomycetemcomitans* from areas of the plaque biofilm and prevent aggregation within the biofilm (original image created by the author for this manuscript with Biorender.com).

The first of these approaches stem from the elegant studies employing novel immunofluorescent techniques to identify multiple pathobionts in the biofilm using a combination of two florescent probes out of a library of individual colors of a defined spectrum [75,76]. These imaging approaches have enabled investigators to develop multicolored maps of the development of bacterial communities in the biofilm [75,77,78] and the phylum to the species level. Of particular interest for the spatial organization of *A. actinomycetemcomitans* at the phylum level is the demonstration of complexes of *A. actinomycetemcomitans* in a defined area consisting of a “hedgehog-shaped” core of corynebacteria connected by *F. nucleatum* [76] (Figure 3). These hedgehogs are studded at the surface by commensals such as streptococcus species and pathobionts such as *P. gingivalis* in a corncob appearance and then surrounded by pathobionts of the same phyla as *A. actinomycetemcomitans*, which may be attached to these corncobs [76,78] (Figure 3).

In this complex structure, the role of *F. nucleatum* is of particular interest, as it appears to serve as a geographical “isthmus” between bacterial species at the core and surface through several types of adhesins [79]. In addition, other images of different sectional views using this staining technique demonstrated the presence of the pathobionts in discrete patches [76]. Since the specifics of *A. actinomycetemcomitans* binding to *F. nucleatum* have not been studied at this molecular level, further studies are required. It is difficult to study *A. actinomycetemcomitans* and its ability to become an early colonizer of teeth in humans [70]. One such study tested for the presence of *A. actinomycetemcomitans* on buccal epithelial cells in volunteers prior to entering them into a longitudinal in vivo study [70]. Six sterile hydroxyapatite squares were placed into an acrylic stent, and one square was removed at intervals ranging from 5 min to 7 h after placement, sonicated, and screened for early colonizing bacteria. *A. actinomycetemcomitans* was found in volunteers 4, 6, and 7 h after placement and only in volunteers with *A. actinomycetemcomitans* prior to square placement. Streptococci and Actinomyces were found at all time points and in both *A. actinomycetemcomitans*-positive and *A. actinomycetemcomitans*-negative volunteers. Fusobacteria were also found at later time points.

Turning to *P. gingivalis* and its effects on the biogeography of *A. actinomycetemcomitans* within the biofilm, a major pathogenic product of *P. gingivalis* is the family of gingipains, which are required for the breakdown of healthy surrounding tissue for essential nutrients [80]. The geographic proximity of *A. actinomycetemcomitans* and *P. gingivalis* as described in the immunofluorescent approaches described above [76] raises interesting questions as to the negative effects of these gingipains on the colonization of *A. actinomycetemcomitans* within the plaque biofilm (Figure 3). While recent studies have shown that these gingipains also have adhesin binding sites to various hard and soft tissue surfaces in the oral cavity [81], in vitro studies have demonstrated that these gingipains can promote the detachment of *A. actinomycetemcomitans* from hard tissue surfaces and an inhibition of the aggregation of *A. actinomycetemcomitans* [82,83]. However, it is important to point out that *P. gingivalis* is a more fastidious anaerobe and occurs at later stages of plaque development [48].

**Figure 4:**
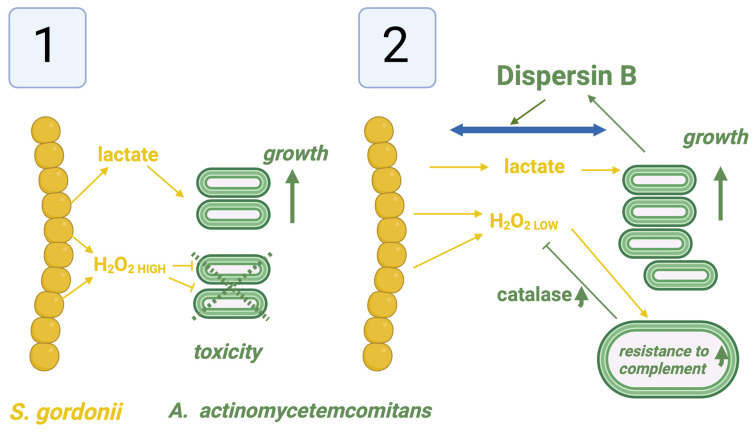
“Fight/Flight interactions between *A. actinomycetemcomitans* and *S. gordonii*. (**1**) At the closest geographical distances between these two microbial species, the beneficial effects of lactate production from *S. gordonii* on the growth of *A actinomycetemcomitans* are offset by the higher toxic concentrations of H_2_O_2_. (**2**) However, the production of dipersin B by *A. actinomycetemcomitans* facilitates a separation from *S. gordonii* and an exposure to a lower and less toxic concentration of H_2_O_2_, which can be neutralized by catalases from *A. actinomycetemcomitans*, stimulate resistance to complement from the host response, and maintain some of the nutritional benefits of *S. gordonii* produced lactate (original image created by the author for this manuscript with Biorender.com).

A second interesting geographical interaction at the micro/nanoscale between two species within biofilms is between *A. actinomycetemcomitans* and *Streptococcus gordonii* (*S. gordonii*) in what has been described as a “fight or flight interaction” (Figure 4). *S. gordonii* can also play a role in the chain of formation of biofilms in the adhesion and colonization of *P. gingivalis* within the biofilm of some periodontal lesions [5,84]. In addition, as noted above, in the absence of gingipains from *P. gingivalis* or other factors, *A. actinomycetemcomitans* may form strong adhesions to saliva-treated surfaces [1] and promote a local pathogenic colony formation of pathobionts within the biofilm [85]. For *A. actinomycetemcomitans*, per se, the close geographical proximity to *S. gordonii* benefits its growth due to the production of lactate by *S. gordonii*, which contributes to the survival and pathogenicity of *A. actinomycetemcomitans* [86,87,88]. However, since *S. gordonii* also produces hydrogen peroxide (H_2_O_2_), which is toxic to *A. actinomycetemcomitans* at higher concentrations, a direct adherence would be potentially toxic to *A. actinomycetemcomitans*. To ensure a safe working distance between these two species, *A. actinomycetemcomitans* produces an enzyme, dispersin B, that promotes movement of this bacteria a short distance away from a higher concentration of this H_2_O_2_ in a “flight” response. *A. actinomycetemcomitans* also produces catalase to neutralize the lower concentrations of H_2_O_2_ at this safer distance [87]. At the same time, during this process, the exposure of *A. actinomycetemcomitans* to H_2_O_2_ produced in part by *S. gordonii* may aid in the formation of surface membrane receptors on *A. actinomycetemcomitans* that increase the resistance to complement-mediated destruction in yet another “fight” response. One additional geographical interaction that is less understood is that of *A. actinomycetemcomitans* and *F. alocis* [74,89,90]. While supported by both in vivo and in vitro studies, these interactions appear to be strain-dependent, and most evidence suggests that *F. alocis*, another leukotoxin producer, appears to occur after *A. actinomycetemcomitans* is present in deep subgingival pockets [91].

## 6. Conclusions

On this tour of microbial geography of *A. actinomycetemcomitans* from the global to nanoscale, we have presented the range of tools used to answer questions regarding (1) the origins and spread of this pathobiont; (2) the establishment, survival, and pathogenicity of this pathobiont; and (3) the complexity of biofilms and other oral microbial communities. Each of these tools can be considered a form of communication, with some common and unique features of grammar, syntax, and vocabulary down to the morphemes and phonemes in the genetic, environmental, and biochemical realm. Questions of the historical origin of *A. actinomycetemcomitans* and its spread to communities around the world and to families and close geographic probands have been answered in part by these approaches. Nevertheless, several central questions remain at a higher magnification level. These include the higher detection rates of *A. actinomycetemcomitans* within those localized areas of first molars and incisors typical of patients with the now defined Localized Stage III Grade C periodontitis and features of the microbiome as a whole in these areas of localized destruction of the periodontal support that can be explained by the variations in the geographical environment within the entire mouth and within the individual microbiomes and biofilms around tooth types and locations. The broad range of newer techniques described in this paper may provide novel hypotheses and answers to these questions. On the whole-mouth scale, a study of the variations in the “climate and ecology” of different geographical environments may provide some answers. In addition, studies that focus on the geographical proximity of *A. actinomycetemcomitans* to other biofilm species, including other pathobionts and commensals, followed by investigations of potential beneficial and biochemical reactions may yield new insights into the role of geography in the role of *A. actinomycetemcomitans* in this unique periodontal disease pattern. While the association of *A. actinomycetemcomitans* in systemic diseases is limited at the present time when compared to the more extensive work with other periodontal pathobionts such as *P. gingivalis*, future studies looking at other possible periodontal disease–systemic links may find that *A. actinomycetemcomitans* possesses important properties that permit translocation and exacerbate systemic diseases, particularly coronary heart diseases.

## Data Availability

Not applicable.

## References

[1] Fine D.H., Schreiner H., Velusamy S.K. (2020). Aggregatibacter, A Low Abundance Pathobiont That Influences Biogeography, Microbial Dysbiosis, and Host Defense Capabilities in Periodontitis: The History of A Bug, And Localization of Disease. Pathogens.

[2] Murray J.L., Connell J.L., Stacy A., Turner K.H., Whiteley M. (2014). Mechanisms of synergy in polymicrobial infections. J. Microbiol..

[3] Kittichotirat W., Bumgarner R.E., Chen C. (2016). Evolutionary Divergence of *Aggregatibacter actinomycetemcomitans*. J. Dent. Res..

[4] Kittichotirat W., Bumgarner R.E., Chen C. (2022). Genomic Islands Shape the Genetic Background of Both JP2 and Non-JP2 *Aggregatibacter actinomycetemcomitans*. Pathogens.

[5] Asikainen S., Chen C., Saarela M., Saxen L., Slots J. (1997). Clonal specificity of *Actinobacillus actinomycetemcomitans* in destructive periodontal disease. Clin. Infect. Dis..

[6] Adsera A., Pytlikova M. (2015). The role of language in shaping international migration. Econ. J..

[7] Nielsen R., Akey J.M., Jakobsson M., Pritchard J.K., Tishkoff S., Willerslev E. (2017). Tracing the peopling of the world through genomics. Nature.

[8] Diamond J.M. (2000). Taiwan’s gift to the world. Nature.

[9] Larson G., Cucchi T., Fujita M., Matisoo-Smith E., Robins J., Anderson A., Rolett B., Spriggs M., Dolman G., Kim T.H. (2007). Phylogeny and ancient DNA of Sus provides insights into neolithic expansion in Island Southeast Asia and Oceania. Proc. Natl. Acad. Sci. USA.

[10] Maier P.A., Runfeldt G., Estes R.J., Vilar M.G. (2022). African mitochondrial haplogroup L7: A 100,000-year-old maternal human lineage discovered through reassessment and new sequencing. Sci. Rep..

[11] Markov P.V., Ghafari M., Beer M., Lythgoe K., Simmonds P., Stilianakis N.I., Katzourakis A. (2023). The evolution of SARS-CoV-2. Nat. Rev. Microbiol..

[12] Tegally H., San J.E., Cotten M., Moir M., Tegomoh B., Mboowa G., Martin D.P., Baxter C., Lambisia A.W., Diallo A. (2022). The evolving SARS-CoV-2 epidemic in Africa: Insights from rapidly expanding genomic surveillance. Science.

[13] Fine D.H., Markowitz K., Furgang D., Fairlie K., Ferrandiz J., Nasri C., McKiernan M., Gunsolley J. (2007). *Aggregatibacter actinomycetemcomitans* and its relationship to initiation of localized aggressive periodontitis: Longitudinal cohort study of initially healthy adolescents. J. Clin. Microbiol..

[14] Haubek D., Ennibi O.K., Poulsen K., Poulsen S., Benzarti N., Kilian M. (2001). Early-onset periodontitis in Morocco is associated with the highly leukotoxic clone of *Actinobacillus actinomycetemcomitans*. J. Dent. Res..

[15] Haubek D., Poulsen K., Kilian M. (2007). Microevolution and patterns of dissemination of the JP2 clone of *Aggregatibacter* (*Actinobacillus*) *actinomycetemcomitans*. Infect. Immun..

[16] Johansson A., Claesson R., Hoglund Aberg C., Haubek D., Lindholm M., Jasim S., Oscarsson J. (2019). Genetic Profiling of *Aggregatibacter actinomycetemcomitans* Serotype B Isolated from Periodontitis Patients Living in Sweden. Pathogens.

[17] Claesson R., Oscarsson J., Johansson A. (2022). Carriage of the JP2 Genotype of *Aggregatibacter actinomycetemcomitans* by Periodontitis Patients of Various Geographic Origin, Living in Sweden. Pathogens.

[18] Jensen A.B., Isidor F., Lund M., Vaeth M., Johansson A., Lauritsen N.N., Haubek D. (2020). Prevalence of *Aggregatibacter actinomycetemcomitans* and Periodontal Findings among 14 to 15-Year Old Danish Adolescents: A Descriptive Cross-Sectional Study. Pathogens.

[19] Meng H., Ren X., Tian Y., Feng X., Xu L., Zhang L., Lu R., Shi D., Chen Z. (2011). Genetic study of families affected with aggressive periodontitis. Periodontol. 2000.

[20] DiRienzo J.M., Slots J., Sixou M., Sol M.A., Harmon R., McKay T.L. (1994). Specific genetic variants of *Actinobacillus actinomycetemcomitans* correlate with disease and health in a regional population of families with localized juvenile periodontitis. Infect. Immun..

[21] Kononen E. (2000). Development of oral bacterial flora in young children. Ann. Med..

[22] Jensen A.B., Ennibi O.K., Ismaili Z., Poulsen K., Haubek D. (2016). The JP2 genotype of *Aggregatibacter actinomycetemcomitans* and marginal periodontitis in the mixed dentition. J. Clin. Periodontol..

[23] Cassini M.A., Pilloni A., Condo S.G., Vitali L.A., Pasquantonio G., Cerroni L. (2013). Periodontal bacteria in the genital tract: Are they related to adverse pregnancy outcome?. Int. J. Immunopathol. Pharmacol..

[24] Dogan B., Kipalev A.S., Okte E., Sultan N., Asikainen S.E. (2008). Consistent intrafamilial transmission of *Actinobacillus actinomycetemcomitans* despite clonal diversity. J. Periodontol..

[25] Tuite-McDonnell M., Griffen A.L., Moeschberger M.L., Dalton R.E., Fuerst P.A., Leys E.J. (1997). Concordance of *Porphyromonas gingivalis* colonization in families. J. Clin. Microbiol..

[26] Lamell C.W., Griffen A.L., McClellan D.L., Leys E.J. (2000). Acquisition and colonization stability of *Actinobacillus actinomycetemcomitans* and *Porphyromonas gingivalis* in children. J. Clin. Microbiol..

[27] Petit M.D., van Steenbergen T.J., Scholte L.M., van der Velden U., de Graaff J. (1993). Epidemiology and transmission of *Porphyromonas gingivalis* and *Actinobacillus actinomycetemcomitans* among children and their family members. A report of 4 surveys. J. Clin. Periodontol..

[28] Monteiro M.F., Altabtbaei K., Kumar P.S., Casati M.Z., Ruiz K.G.S., Sallum E.A., Nociti-Junior F.H., Casarin R.C.V. (2021). Parents with periodontitis impact the subgingival colonization of their offspring. Sci. Rep..

[29] Koo S.S., Fernandes J.G., Li L., Huang H., Aukhil I., Harrison P., Diaz P.I., Shaddox L.M. (2024). Evaluation of microbiome in primary and permanent dentition in grade C periodontitis in young individuals. J. Periodontol..

[30] Haffajee A.D., Patel M., Socransky S.S. (2008). Microbiological changes associated with four different periodontal therapies for the treatment of chronic periodontitis. Oral Microbiol. Immunol..

[31] Shiloah J., Patters M.R. (1996). Repopulation of periodontal pockets by microbial pathogens in the absence of supportive therapy. J. Periodontol..

[32] Gunsolley J.C., Zambon J.J., Mellott C.A., Brooks C.N., Kaugars C.C. (1994). Maintenance therapy in young adults with severe generalized periodontitis. J. Periodontol..

[33] Kuraji R., Sekino S., Kapila Y., Numabe Y. (2021). Periodontal disease-related nonalcoholic fatty liver disease and nonalcoholic steatohepatitis: An emerging concept of oral-liver axis. Periodontol. 2000.

[34] Ryder M.I., Xenoudi P. (2021). Alzheimer disease and the periodontal patient: New insights, connections, and therapies. Periodontol. 2000.

[35] Gualtero D.F., Lafaurie G.I., Buitrago D.M., Castillo Y., Vargas-Sanchez P.K., Castillo D.M. (2023). Oral microbiome mediated inflammation, a potential inductor of vascular diseases: A comprehensive review. Front. Cardiovasc. Med..

[36] de Jongh C.A., de Vries T.J., Bikker F.J., Gibbs S., Krom B.P. (2023). Mechanisms of *Porphyromonas gingivalis* to translocate over the oral mucosa and other tissue barriers. J. Oral Microbiol..

[37] Kajiya M., Komatsuzawa H., Papantonakis A., Seki M., Makihira S., Ouhara K., Kusumoto Y., Murakami S., Taubman M.A., Kawai T. (2011). *Aggregatibacter actinomycetemcomitans* Omp29 is associated with bacterial entry to gingival epithelial cells by F-actin rearrangement. PLoS ONE.

[38] Yuan A., Yang P.C., Lee L.N., Chang D.B., Kuo S.H., Luh K.T. (1992). *Actinobacillus actinomycetemcomitans* pneumonia with chest wall involvement and rib destruction. Chest.

[39] Kaplan A.H., Weber D.J., Oddone E.Z., Perfect J.R. (1989). Infection due to *Actinobacillus actinomycetemcomitans*: 15 cases and review. Rev. Infect. Dis..

[40] Paju S., Carlson P., Jousimies-Somer H., Asikainen S. (2000). Heterogeneity of *Actinobacillus actinomycetemcomitans* strains in various human infections and relationships between serotype, genotype, and antimicrobial susceptibility. J. Clin. Microbiol..

[41] Paturel L., Casalta J.P., Habib G., Nezri M., Raoult D. (2004). *Actinobacillus actinomycetemcomitans* endocarditis. Clin. Microbiol. Infect..

[42] Tang G., Kitten T., Munro C.L., Wellman G.C., Mintz K.P. (2008). EmaA, a potential virulence determinant of *Aggregatibacter actinomycetemcomitans* in infective endocarditis. Infect. Immun..

[43] Reyes L., Herrera D., Kozarov E., Rolda S., Progulske-Fox A. (2013). Periodontal bacterial invasion and infection: Contribution to atherosclerotic pathology. J. Periodontol..

[44] Mark Welch J.L., Dewhirst F.E., Borisy G.G. (2019). Biogeography of the Oral Microbiome: The Site-Specialist Hypothesis. Annu. Rev. Microbiol..

[45] Bik E.M., Long C.D., Armitage G.C., Loomer P., Emerson J., Mongodin E.F., Nelson K.E., Gill S.R., Fraser-Liggett C.M., Relman D.A. (2010). Bacterial diversity in the oral cavity of 10 healthy individuals. ISME J..

[46] Segata N., Haake S.K., Mannon P., Lemon K.P., Waldron L., Gevers D., Huttenhower C., Izard J. (2012). Composition of the adult digestive tract bacterial microbiome based on seven mouth surfaces, tonsils, throat and stool samples. Genome Biol..

[47] Claesson R., Johansson A., Belibasakis G.N. (2023). Age-Related Subgingival Colonization of *Aggregatibacter actinomycetemcomitans*, *Porphyromonas gingivalis* and *Parvimonas micra*—A Pragmatic Microbiological Retrospective Report. Microorganisms.

[48] Socransky S.S., Haffajee A.D., Ximenez-Fyvie L.A., Feres M., Mager D. (1999). Ecological considerations in the treatment of *Actinobacillus actinomycetemcomitans* and *Porphyromonas gingivalis* periodontal infections. Periodontol. 2000.

[49] Zhang Z., Liu D., Liu S., Zhang S., Pan Y. (2020). The Role of *Porphyromonas gingivalis* Outer Membrane Vesicles in Periodontal Disease and Related Systemic Diseases. Front. Cell. Infect. Microbiol..

[50] Ha J.Y., Seok J., Kim S.J., Jung H.J., Ryu K.Y., Nakamura M., Jang I.S., Hong S.H., Lee Y., Lee H.J. (2023). Periodontitis promotes bacterial extracellular vesicle-induced neuroinflammation in the brain and trigeminal ganglion. PLoS Pathog..

[51] Pritchard A.B., Fabian Z., Lawrence C.L., Morton G., Crean S., Alder J.E. (2022). An Investigation into the Effects of Outer Membrane Vesicles and Lipopolysaccharide of *Porphyromonas gingivalis* on Blood-Brain Barrier Integrity, Permeability, and Disruption of Scaffolding Proteins in a Human in vitro Model. J. Alzheimer’s Dis..

[52] Dominy S.S., Lynch C., Ermini F., Benedyk M., Marczyk A., Konradi A., Nguyen M., Haditsch U., Raha D., Griffin C. (2019). *Porphyromonas gingivalis* in Alzheimer’s disease brains: Evidence for disease causation and treatment with small-molecule inhibitors. Sci. Adv..

[53] Slots J., Feik D., Rams T.E. (1990). *Actinobacillus actinomycetemcomitans* and *Bacteroides intermedius* in human periodontitis: Age relationship and mutual association. J. Clin. Periodontol..

[54] Haffajee A.D., Teles R.P., Patel M.R., Song X., Yaskell T., Socransky S.S. (2009). Factors affecting human supragingival biofilm composition. II. Tooth position. J. Periodontal Res..

[55] Simon-Soro A., Tomas I., Cabrera-Rubio R., Catalan M.D., Nyvad B., Mira A. (2013). Microbial geography of the oral cavity. J. Dent. Res..

[56] Haffajee A.D., Socransky S.S., Smith C., Dibart S., Goodson J.M. (1992). Subgingival temperature (III). Relation to microbial counts. J. Clin. Periodontol..

[57] Proctor D.M., Fukuyama J.A., Loomer P.M., Armitage G.C., Lee S.A., Davis N.M., Ryder M.I., Holmes S.P., Relman D.A. (2018). A spatial gradient of bacterial diversity in the human oral cavity shaped by salivary flow. Nat. Commun..

[58] Dawes C., Watanabe S., Biglow-Lecomte P., Dibdin G.H. (1989). Estimation of the velocity of the salivary film at some different locations in the mouth. J. Dent. Res..

[59] Haffajee A.D., Teles R.P., Patel M.R., Song X., Veiga N., Socransky S.S. (2009). Factors affecting human supragingival biofilm composition. I. Plaque mass. J. Periodontal Res..

[60] Proctor D.M., Shelef K.M., Gonzalez A., Davis C.L., Dethlefsen L., Burns A.R., Loomer P.M., Armitage G.C., Ryder M.I., Millman M.E. (2020). Microbial biogeography and ecology of the mouth and implications for periodontal diseases. Periodontol. 2000.

[61] Proctor D.M., Relman D.A. (2017). The Landscape Ecology and Microbiota of the Human Nose, Mouth, and Throat. Cell Host Microbe.

[62] Sedghi L., DiMassa V., Harrington A., Lynch S.V., Kapila Y.L. (2021). The oral microbiome: Role of key organisms and complex networks in oral health and disease. Periodontol. 2000.

[63] Shaddox L.M., Huang H., Lin T., Hou W., Harrison P.L., Aukhil I., Walker C.B., Klepac-Ceraj V., Paster B.J. (2012). Microbiological characterization in children with aggressive periodontitis. J. Dent. Res..

[64] Velsko I.M., Harrison P., Chalmers N., Barb J., Huang H., Aukhil I., Shaddox L. (2020). Grade C molar-incisor pattern periodontitis subgingival microbial profile before and after treatment. J. Oral Microbiol..

[65] Amado P.P.P., Kawamoto D., Albuquerque-Souza E., Franco D.C., Saraiva L., Casarin R.C.V., Horliana A., Mayer M.P.A. (2020). Oral and Fecal Microbiome in Molar-Incisor Pattern Periodontitis. Front. Cell. Infect. Microbiol..

[66] Mombelli A., Meier C. (2001). On the symmetry of periodontal disease. J. Clin. Periodontol..

[67] Haffajee A.D., Socransky S.S., Smith C., Dibart S. (1992). The use of DNA probes to examine the distribution of subgingival species in subjects with different levels of periodontal destruction. J. Clin. Periodontol..

[68] Clarridge J.E. (2004). Impact of 16S rRNA gene sequence analysis for identification of bacteria on clinical microbiology and infectious diseases. Clin. Microbiol. Rev..

[69] Baer P.N. (1971). The case for periodontosis as a clinical entity. J. Periodontol..

[70] Fine D.H., Markowitz K., Furgang D., Velliyagounder K. (2010). *Aggregatibacter actinomycetemcomitans* as an early colonizer of oral tissues: Epithelium as a reservoir?. J. Clin. Microbiol..

[71] Hajishengallis G., Lamont R.J. (2016). Dancing with the Stars: How Choreographed Bacterial Interactions Dictate Nososymbiocity and Give Rise to Keystone Pathogens, Accessory Pathogens, and Pathobionts. Trends Microbiol..

[72] Kolenbrander P.E., Palmer R.J., Rickard A.H., Jakubovics N.S., Chalmers N.I., Diaz P.I. (2006). Bacterial interactions and successions during plaque development. Periodontol. 2000.

[73] Moustafa A.M., Velusamy S.K., Denu L., Narechania A., Fine D.H., Planet P.J. (2021). Adaptation by Ancient Horizontal Acquisition of Butyrate Metabolism Genes in *Aggregatibacter actinomycetemcomitans*. mBio.

[74] Fine D.H., Markowitz K., Fairlie K., Tischio-Bereski D., Ferrendiz J., Furgang D., Paster B.J., Dewhirst F.E. (2013). A consortium of *Aggregatibacter actinomycetemcomitans*, *Streptococcus parasanguinis*, and *Filifactor alocis* is present in sites prior to bone loss in a longitudinal study of localized aggressive periodontitis. J. Clin. Microbiol..

[75] Valm A.M., Mark Welch J.L., Rieken C.W., Hasegawa Y., Sogin M.L., Oldenbourg R., Dewhirst F.E., Borisy G.G. (2011). Systems-level analysis of microbial community organization through combinatorial labeling and spectral imaging. Proc. Natl. Acad. Sci. USA.

[76] Mark Welch J.L., Rossetti B.J., Rieken C.W., Dewhirst F.E., Borisy G.G. (2016). Biogeography of a human oral microbiome at the micron scale. Proc. Natl. Acad. Sci. USA.

[77] Ramirez-Puebla S.T., Mark Welch J.L., Borisy G.G. (2024). Improved Visualization of Oral Microbial Consortia. J. Dent. Res..

[78] Morillo-Lopez V., Sjaarda A., Islam I., Borisy G.G., Mark Welch J.L. (2022). Corncob structures in dental plaque reveal microhabitat taxon specificity. Microbiome.

[79] Groeger S., Zhou Y., Ruf S., Meyle J. (2022). Pathogenic Mechanisms of *Fusobacterium nucleatum* on Oral Epithelial Cells. Front. Oral Health.

[80] Potempa J., Banbula A., Travis J. (2000). Role of bacterial proteinases in matrix destruction and modulation of host responses. Periodontol. 2000.

[81] Chen T., Nakayama K., Belliveau L., Duncan M.J. (2001). *Porphyromonas gingivalis* gingipains and adhesion to epithelial cells. Infect. Immun..

[82] Haraguchi A., Miura M., Fujise O., Hamachi T., Nishimura F. (2014). *Porphyromonas gingivalis* gingipain is involved in the detachment and aggregation of *Aggregatibacter actinomycetemcomitans* biofilm. Mol. Oral Microbiol..

[83] Takasaki K., Fujise O., Miura M., Hamachi T., Maeda K. (2013). *Porphyromonas gingivalis* displays a competitive advantage over *Aggregatibacter actinomycetemcomitans* in co-cultured biofilm. J. Periodontal Res..

[84] Wright C.J., Burns L.H., Jack A.A., Back C.R., Dutton L.C., Nobbs A.H., Lamont R.J., Jenkinson H.F. (2013). Microbial interactions in building of communities. Mol. Oral Microbiol..

[85] Planet P.J., Kachlany S.C., Fine D.H., DeSalle R., Figurski D.H. (2003). The Widespread Colonization Island of *Actinobacillus actinomycetemcomitans*. Nat. Genet..

[86] Brown S.A., Whiteley M. (2007). A novel exclusion mechanism for carbon resource partitioning in *Aggregatibacter actinomycetemcomitans*. J. Bacteriol..

[87] Stacy A., Everett J., Jorth P., Trivedi U., Rumbaugh K.P., Whiteley M. (2014). Bacterial fight-and-flight responses enhance virulence in a polymicrobial infection. Proc. Natl. Acad. Sci. USA.

[88] Ramsey M.M., Rumbaugh K.P., Whiteley M. (2011). Metabolite cross-feeding enhances virulence in a model polymicrobial infection. PLoS Pathog..

[89] Razooqi Z., Tjellstrom I., Hoglund Aberg C., Kwamin F., Claesson R., Haubek D., Johansson A., Oscarsson J. (2024). Association of *Filifactor alocis* and its RTX toxin gene ftxA with periodontal attachment loss, and in synergy with *Aggregatibacter actinomycetemcomitans*. Front. Cell. Infect. Microbiol..

[90] Wang Q., Wright C.J., Dingming H., Uriarte S.M., Lamont R.J. (2013). Oral community interactions of *Filifactor alocis* in vitro. PLoS ONE.

[91] Oscarsson J., Claesson R., Bao K., Brundin M., Belibasakis G.N. (2020). Phylogenetic Analysis of *Filifactor alocis* Strains Isolated from Several Oral Infections Identified a Novel RTX Toxin, FtxA. Toxins.

